# Nanotechnology-Based Histone Deacetylase Inhibitors for Cancer Therapy

**DOI:** 10.3389/fcell.2020.00400

**Published:** 2020-06-03

**Authors:** Bin Tu, Meng Zhang, Tuanbing Liu, Yongzhuo Huang

**Affiliations:** ^1^State Key Laboratory of Drug Research, Shanghai Institute of Materia Medica, Chinese Academy of Sciences, Shanghai, China; ^2^University of Chinese Academy of Sciences, Beijing, China; ^3^NMPA Key Laboratory for Quality Research and Evaluation of Pharmaceutical Excipients, Beijing, China

**Keywords:** Histone deacetylase inhibitors (HDACi), nanotechnology, nanomedicine, cancer therapy, solid tumor, targeting drug delivery, combination therapy

## Abstract

Histone deacetylase inhibitors (HDACi) have been approved and achieved success in hematologic malignancies. But its application in solid tumors still confronts big challenges and is hampered by low treatment efficacy. Nanotechnology has been widely applied in cancer therapy, and nanomedicine could improve drug stability, prolong the circulation half-life, and increase intratumoral drug accumulation. Therefore, nanomedicine is a promising strategy to enhance HDACi therapy efficacy. The review provides a summary of the advances of HDACi nanomedicines with a focus on the design principles of the targeting delivery systems for HDACi.

## Introduction

**FIGURE 1:**
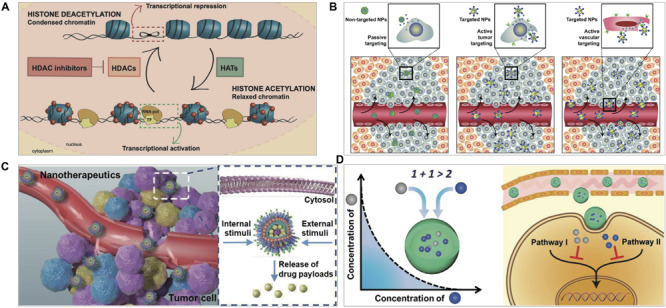
**(A)** HDACi effect on chromatin remodeling. HDACs deacetylate histones and inhibit gene transcription and histone acetyltransferases (HATs) acetylate histones and activate transcription. HDACi inhibit HDACs, and thus maintain an open chromatin conformation (San Jose-Eneriz et al., 2019). **(B)** Nanotechnology-based targeting delivery (Farokhzad and Langer, 2009). **(C)** Stimuli-responsive controlled release (Qiao et al., 2019). **(D)** Nanotechnology-based combination (Ma et al., 2013). Figures are reproduced with permission from the publishers of the cited references.

Histone deacetylase inhibitors (HDACi) prevent histone deacetylases from removing acetyl group from the lysine amino acid of histone and thus open the chromatin structure to promote the access to transcription factors and facilitate gene transcription, consequently regulating cell proliferation (Figure 1A). HDACi exert anticancer mechanisms of HDACi include cell cycle arrest, apoptosis, autophagy, anti-angiogenesis, and regulation of epithelial-mesenchymal transition and immune responses (Eckschlager et al., 2017; Wawruszak et al., 2019). United States FDA has approved five HDACi for hematologic malignancies, including vorinostat (2006), romidepsin (2009), belinostat (2014), chidamide (2015), and panobinostat (2015).

However, there is limited success in solid tumor treatment, which could account for various reasons. First, treatment efficacy is affected by the poor pharmacokinetics such as short half-life, and fast metabolism and clearance; second, low specificity often causes off-target and side effects; third, low solubility and tissue/cell permeability of HDACi limit intratumor delivery; and fouth, drug resistance is readily developed (Li and Seto, 2016; Suraweera et al., 2018). Targeted delivery and controlled drug release could be a potential solution to these issues in HDACi-based therapy.

Nanomedicine is referred to as a specific size – “about 100 nanometers or less – that biological molecules and structures operate in living cells” (NIH), but often referred to a wider scale – less than 1000 nm. Cancer nanomedicines can improve efficacy and reduce side-toxicity by increasing drug solubility, improving the pharmacokinetic profiles, and enhancing intratumoral drug delivery (Bayda et al., 2018; Palazzolo et al., 2018).

For example, rational design of nanoparticles can enhance drug accumulation and cellular uptake in the tumor by fine-tuning size and surface property. Small size can increase intratumoral infiltration because nanoparticles with size between 20 and 60 nm are more likely to penetrate deep tumor tissues via the enhanced permeability and retention (EPR) effect and uptaken by tumor cells, while the larger is easily captured by the reticuloendothelial system (RES) and the smaller less than 5 nm cleared by renal (Bayda et al., 2018).

## Nanotechnology-Based Platforms of Hdaci for Cancer Therapy

There are two strategies for tumor-targeting delivery – passive and active targeting. Nanomedicine could preferentially accumulate into the tumors to achieve passive targeting through EPR effect, by which the macromolecules or nanomaterials tend to distribute in the tumor due to the leaky neovasculature and poor lymphatic drainage (Maeda, 2012; Ahmad et al., 2019). EPR effect is typically observed in rodent tumor models, but with limited evidence in human patients. It is believed that the EPR effect is highly variable due to the heterogeneity of the tumors (Danhier, 2016). Interestingly, a recent study found that 97% of PEGylated AuNPs entered tumors via active *trans-*endothelial pathways, while inter-endothelial gaps accounted for a very small proportion (Sindhwani et al., 2020). However, the conclusion was drawn based on the non-deformable AuNPs and needs further support by using various nanoparticles including the deformable vesicles.

Active-targeting delivery represents another common strategy. The general design is based on ligand/receptor-specific interaction (e.g., trastuzumab and HER-2, folate and folate receptors): the modified ligands on nanomedicine and the receptors expressed on cancer cells or other cells in tumor microenvironment (TME) (Figure 1B). Certain nanomaterials can selectively bind with the receptors on cancer cells, e.g., the interaction of albumin nanoparticles and albumin-binding proteins (Lin et al., 2016; Zhao et al., 2018a).

### Nanotechnology-Based Passive Targeting

Tumor vessels are characterized by the abnormal vasculatures with the leaky vessel walls that lack tight junction between endothelial cells and pericytes. The leakage facilitates nanomedicine to permeate and accumulate in the tumor tissues, compared to the normal tissues with 5–10 nm endothelial junctions (Hobbs et al., 1998). The preferential accumulation of drugs in the tumor means the lower drug dose and fewer side effects. Moreover, nanomedicines can increase the intracellular drug retention based on the “size-exclusion effect” that reduces the transporters-mediated drug efflux and reverse drug resistance (Liu et al., 2012), because P-glycoproteins can only recognize the substrate molecules less than 2000 Da (Seelig, 1998).

Nanotechnology-based delivery can improve anti-tumor efficacy of HDACi by overcoming their disadvantages. For example, poor solubility is a problem for many HDACi. Starch is a polysaccharide widely used in pharmaceutics due to its biocompatibility and biodegradability. Encapsulating CG-1521 (an HDACi) into the starch nanoparticles via the emulsion-solvent diffusion method could increase its water solubility and cellular uptake, leading to enhanced anti-tumor activity (Alp et al., 2019). Panobinostat has been explored for glioma treatment. However, the poor solubility prevents its administration by convection-enhanced delivery (CED) in brain cancer. To solve this problem, an HDACi nano-system composed of poloxamer 407 (P407) was designed (Singleton et al., 2017). P407 can self-assemble into nano-micelles based on its hydrophobic polypropylene glycol chain in the middle and two hydrophilic PEG chains at both ends. The panobinostat-loaded P407 micelles increased the intracranial panobinostat concentration by skirting the blood-brain barrier (BBB), resulting in glioma repression and prolonged survival of the high-grade glioma-bearing rats by one CED dose.

Poly(D, L-lactide-co-glycolide) (PLGA) is an injectable polymer approved by FDA. The PEG-lecithin coated PLGA nanoparticles encapsulating vorinostat or quisinostat were prepared via the single-step nanoprecipitation method; the delivery systems achieved the controlled release and suppressed the DNA double-strand break (DSB) repair in tumor cells, serving as the potential radiosensitizers and exhibiting synergistic effect in the mouse xenograft models of colorectal and prostate carcinomas via a mechanism of the prolongation of γ-H2AX foci (Wang et al., 2015).

Owing to the existence of the bladder permeability barrier (BPB), drugs are difficult to distribute in the bladder tumor. The nano delivery strategy through the EPR effect could not help much. To address this problem, poly(guanidinium oxanorbornene) (PGON), a novel cell-penetrating polymer, was used to modify the belinostat-loaded PLGA nanoparticles to improve the BPB-permeability (Martin et al., 2013). The PGON-modified nanoparticles enhanced the penetration in the mouse bladder more than 10 folds compared to the nanoparticles without PGON, and significantly enhanced intracellular uptake and anti-tumor activity.

Furthermore, it has been reported that the biodegradable poly(DL-lactide-co-glycolide)/poly(ethylene glycol) (LGE) block copolymer could encapsulate vorinostat by using the nanoprecipitation method (Kwak et al., 2015). The vorinostat-loaded nanoparticles promoted the intensive accumulation in the tumors and exhibited the enhanced antitumor activity in the subcutaneous cholangiocarcinoma-bearing mice compared to free vorinostat.

### Nanotechnology-Based Active Targeting

There have been various active-targeting nanoparticles developed for HDACi therapy. Ligand-modified nanoparticles facilitate the tumor-targeting and cell internalization via specific interaction between the ligand decorated on nanoparticles and the corresponding receptors overexpressed on the tumor cells. For example, the nutrient transporters (e.g., albumin-binding protein) are often overexpressed in tumor cells due to their hunger for energy and thus serve as the targeting delivery receptors (Zhao et al., 2018b). The active targeting ligand molecules mainly include small molecules, peptides, antibodies, and aptamers (Bazak et al., 2015).

#### Small Molecular Ligand-Mediated Active Targeting

Small molecules are advantageous for their low price, small size, and chemical stability. For example, tumor-associated macrophages (TAM) overexpress mannose receptors and are ideal targets for cancer treatment. Mannose is a specific ligand of the mannose receptor and can be chemically modified to the surface of liposomes to deliver vorinostat for targeting TAM (Peng et al., 2017). Triple-negative breast cancer (TNBC) cells often overexpress lysophosphatidic acid receptor 1 (LPAR1) and G2A receptors. By modification with LPA (a ligand of LPAR1) and LPC (a ligand of G2A), the PEGylated nanoemulsions of panobinostat and decitabine were prepared via the solvent injection method (Kim et al., 2019). The cellular uptake and *in vivo* biodistribution of the nanoemulsions was associated with the LPAR1 expression and the optimal modification ratio of LPA to LPC was 1:1. Furthermore, the folic acid (FA)-modified dendrimer-HDACi conjugates were prepared by click reaction between the azido-modified suberoylanilide hydroxamic acid (SAHA) and polyamidoamine (PAMAM) (Zong et al., 2015). FA mediated tumor-targeting delivery of the conjugates and SAHA remained inactivity until released from the conjugates in the cancer cells.

#### Peptide/Protein Ligand-Mediated Active Targeting

Peptides and proteins are the commonly used ligands. For example, transferrin receptors (TfR) are often overexpressed in many malignant cells for iron uptake (Lai et al., 2009; Amreddy et al., 2018), which is a useful target for cancer drug delivery. A TfR-targeted lipid-protein hybrid nano-platform was designed by encapsulating the vorinostat/paclitaxel co-loading albumin nanoparticles into the transferrin-modified liposomes (Ruttala et al., 2017). The liposomes effectively protected the drugs from rapid elimination during the circulation and the surface-anchored transferrin could bind with TfR on the cancer cells with 8–10 folds of higher efficiency compared with non-targeted nanoparticles. The nano-platform yielded enhanced efficacy in a HepG-2 xenografted mouse model via vorinostat-sensitized paclitaxel chemotherapy.

#### Antibody Ligand-Mediated Active Targeting

**FIGURE 2:**
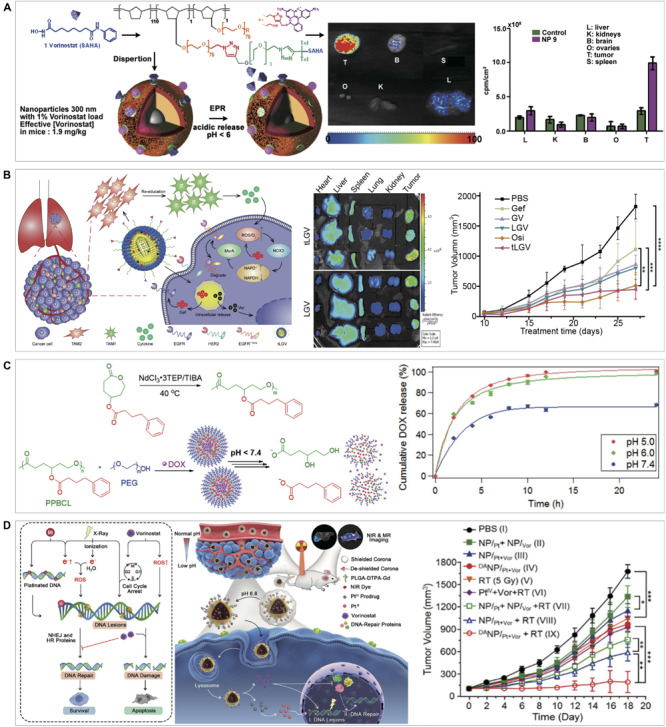
Application examples of nanotechnology-based HDACi therapy. **(A)** Passive targeting: the scheme of vorinostat-polymer conjugate nanoparticles and the enhanced tumor accumulation via the EPR effect (Denis et al., 2014). **(B)** Active targeting: the scheme of dual-targeted liposome system co-loading gefitinib and vorinostat and targeting HER-2 expressed tumor cells and mannose receptor expressed TAMs, its anti-tumor effect on H1975 tumor mouse model, and the accumulation by *in vivo* imaging study (Peng et al., 2017). **(C)** Stimuli-responsive controlled drug release: the illustration of pH-responsive DOX loading nanoparticles by the self-assembling of PBA conjugated polycaprolactone, and the drug release in response to different pH (Kularatne et al., 2018). **(D)** Combination therapy: the scheme of vorinostat-containing nanoparticles for sensitizing radiotherapy (Jiang et al., 2018). *p < 0.05 was considered statistically significant in all analysis (95% confidence level). *p < 0.05, **p < 0.01, ***p < 0.001, and ****p < 0.0001. Figures are reproduced with permission from the publishers of the cited references.

EGFR^T790M^ secondary mutation is readily developed in those receiving gefitinib therapy in non-small cell lung cancer (NSCLC), thus resulting in gefitinib resistance. A dual-targeted liposome system for codelivery of gefitinib and vorinostat has been prepared by chemical modification with anti-HER-2 antibody and mannose (Peng et al., 2017). The liposomes can target the HER-2-overexpressing tumor cells and mannose receptor-expressed TAM, respectively. The dual-targeted liposomes reversed the resistance of EGFR^T790M^-positive NSCLC to gefitinib via reprogramming the TAMs and regulating the ROS/NOX3/MsrA axis of cancer cells (Figure 2B) (Peng et al., 2017).

### Stimuli-Responsive Controlled Drug Release

After nanomedicine reaches the tumor site, the effectively controlled release of drugs becomes a key issue. The drug release rate can significantly affect therapeutic efficacy. For example, fast intracellular drug release was beneficial to overcome chemoresistance and kill the tumor cells (Gao et al., 2011). The stimuli-responsive strategy provides a useful method for achieving site-specific controlled drug release.

Tumor microenvironment is characterized by its abnormal conditions, like acidic pH, enhanced ROS or GSH, and overexpressed proteases, which serve as endogenous triggers to stimulate drug release from nanomedicines (Chen et al., 2016). Besides, exogenous stimuli can also be used for controlled drug release, such as light, ultrasound, and external alternating magnetic field (Patra et al., 2018). Various stimuli-responsive nanomedicines have been developed for triggering drug release at the tumor (Figure 1C).

#### Redox-Responsive Nanoparticles

There are excessive GSH and/or ROS in cancer cells (Weinberg et al., 2019). Disulfide linkage that can be cleaved by GSH via reduction reaction has been widely used in stimuli-responsive delivery. An amphiphilic prodrug SAHA-S-S-VE was prepared via conjugation of vorinostat (SAHA) with vitamin E (VE) through a disulfide bond, which could self-assemble into the nanoparticles with addition of D-a-tocopheryl polyethylene glycol succinate (TPGS). The prodrug nanoparticles could release vorinostat in response to GSH stimulation in a concentration-dependent manner and showed a significant anti-tumor effect in the mice bearing H22 tumors (Han et al., 2016).

#### pH-Responsive Nanoparticles

The acidic pH ranges from 5–7 in TME caused by the Warburg effect (Dai et al., 2017). Bertrand et al. proposed an interesting ROMP (Ring-Opening Metathesis Polymerization) method for preparing a stealth polymeric nanoparticle platform for delivering HDACi (Bertrand et al., 2019). The alkyne-modified cancer drugs can be grafted to the nanoparticles by click chemistry and the chemical bond can be cleaved by acidic pH, thus achieving enhanced intratumor accumulation and controlled release (Figure 2A) (Denis et al., 2014). The γ-4-phenylbutyrate-ε-caprolactone monomer was synthesized via modifying the 4-phenyl butyric acid (PBA, an HDACi) with a pH-sensitive ester bond and then polymerized to the homopolymer of poly(γ-4-phenylbutyrate-ε-caprolactone) (PPBCL) which could self-assemble into nanoparticles with PEG as a surfactant to encapsulate doxorubicin (DOX) (Kularatne et al., 2018). In the acidic environment, the ester bonds were hydrolyzed and the nanoparticles disintegrated and PBA and DOX released (Figure 2C) (Kularatne et al., 2018). Another study showed that various HDACi prodrugs were prepared via linking n-dodecanoic acid or cholesterol to K-182, a potent HDACi, with ester bond or disulfide bond; the HDACi prodrugs and a DNA drug were incorporated into the cation nanoparticles, and the hyperacetylation of core histones facilitated the enhanced gene expression (Ishii et al., 2009).

#### Enzyme-Responsive Nanoparticles

The overexpressed enzymes in TME are usually associated with cancer progressions, such as matrix metalloproteinases (MMPs), phospholipase A2, and esterase (Fouladi et al., 2017). A typical design is to use an enzyme-substrate sequence or material to conjugate or encapsulate drugs; once cleavage by the tumor-associated enzymes, the drugs would be released. For instance, a hyaluronic acid (HA) derivative (HAPBA) was synthesized via conjugating PBA to HA via ester bonds that are susceptible to esterase (Lee et al., 2019). The HAPBA-based nanoparticles increased intratumoral drug accumulation and capture by tumor cells via endocytosis mediated by CD44, the receptor of HA. The intracellular PBA release was triggered by esterase.

#### Temperature-Responsive Nanogel

A thermosensitive nanogel system composed of poly(ethylene glycol)-poly(e-caprolactone)-poly(ethylene glycol) (PEG-PCL-PEG, PECE) was designed to treat oral squamous cell carcinoma (OSCC) via co-delivering vorinostat and cisplatin (Li et al., 2012). The PECE solution turned into a gel state at body temperature after intratumoral injection. With PECE biodegradation via hydrolysis, drugs were continuously released from the nanogel, yielding enhanced therapeutic effects and reduced side effects.

### Nanotechnology-Based Combination Therapy

Drug combinations can achieve improved therapeutic efficacy with the benefits of reducing side toxicity and drug resistance. HDACi in combination with other anticancer agents have been applied and shown great potential for cancer therapy as a promising solution to the insufficient efficacy of HDACi in solid tumors (Yuan et al., 2017; Suraweera et al., 2018). Yet, a big challenge is the different *in vivo* fate of the combined drugs; for example, the asynchronous distribution in the tumor may not cause synergistic pharmacological actions in the cancer cells (Zhang et al., 2017). It may be responsible for the inconsistency between the *in vitro* and *in vivo* results; many combinations work well in cell tests, but not *in vivo*. Nanotechnology-based combination therapy can achieve the synchronized pharmacokinetic profile of the combined drugs and facilitate the synchronized delivery (Figure 1D), and has been explored in HDACi therapy.

A prodrug conjugate (VAAP) composed of platinum (IV) prodrug (diaminedichlorodihydroxyplatinum, ACHP) and valproic acid (VA, an HDACi) was synthesized for cancer combination therapy (Yang et al., 2012). VAAP was then loaded into the polyethylene glycol-polycaprolactone (PEG-PCL) micelles. Free Pt (II) and VA were released from the VAAP in cancer cells, with 50–100 times higher cytotoxicity than the simple mixture of ACHP and VA. The PEG-PCL/VAAP micelles prolonged drug circulation, and increased intratumoral drug concentration and treatment efficacy in the mice bearing A549 tumors.

A nanomedicine for codelivery of vorinostat and cis, cis, *trans-*[Pt(NH3)_2_Cl_2_(OOC(CH2)_8_CH3)_2_] was developed, which was characterized by the de-shieldable corona that reduced the capture by the RES but was removed in the acidic TME to expose the cell-penetrating peptides (Jiang et al., 2018). The cell-penetrating peptide facilitated the nanomedicine into the tumor cells and the released Pt chelated into DNA and the released vorinostat sensitized the cancer cells by promoting ROS production and inhibiting DAN repair proteins (Figure 2D). Therefore, the nanotechnology-based codelivery yielded a synergistic effect on radiotherapy sensitization.

Another combination therapy strategy is to co-administrate the single drug-loaded nanoparticles with a benefit of fine-tuned proportion. The nanogels were prepared from the polymerization of poly(ethylene glycol) monomethacrylate (POEOMA) to encapsulate vorinostat and etoposide, respectively (Kumar et al., 2018). Both nanogels could release their drugs in response to GSH due to disulfide bond cleavage. The co-administration of vorinostat-loaded nanogel and etoposide-loaded nanogel showed enhanced cytotoxicity in cancer cells.

## Perspectives

Histone deacetylase inhibitors therapy has achieved great success in hematologic malignancies, but with limited progress in solid tumors. Nanotechnology can improve HDACi efficacy by optimizing drug delivery and controlling drug release. For example, tumor-targeting and stimuli-responsive nanomedicines can be designed to increase the intratumoral accumulation and control drug release in the tumors. HDACi nanomedicines showed an improved anti-tumor efficacy with reduced systemic toxicity. Besides, nanotechnology has also applied to predict the therapeutic efficacy of HDACi and a gold nanoparticle nanosensor was developed for the detection of histone deacetylase (Zhang et al., 2019).

However, nanotechnology-based HDACi still confront several challenges. First, nano delivery systems are inclined to be cleared away by RES, often leading to less than 1% of the injected nanoparticles reaching to the solid tumors (Wilhelm et al., 2016). The multi-functional targeting strategies will be helpful to overcome the various biological barriers and further increase intratumoral drug accumulation. Because HDACi act in the nucleus, nanotechnology-based nuclear-targeted delivery strategies would enhance treatment efficacy. Second, stimuli-responsive strategies rely on abnormal elements (e.g., acidic pH) in TME. However, in the highly heterogenetic tumors in the human body, the clinical applicability of such endogenous triggers is still unknown and has not been well investigated. Exogenous stimuli (e.g., light, radiofrequency, and ultrasound) could be more controllable than endogenous triggers. The light-triggered release has lots of reports, but its major problem is poor penetration of light in the human body, rendering limited use in deep tumors such as brain and lung cancers. Radiofrequency and ultrasound are more clinically translational due to the advantages of excellent penetration and energy focus. Yet, Phase III HEAT study using radiofrequency ablation with Lyso-Thermosensitive Liposomal Doxorubicin failed to reach the expected endpoint (Tak et al., 2018). Later, the research team substituted the radiofrequency with an ultrasound trigger for the thermosensitive liposomal doxorubicin and conducted a phase I clinical trial (Lyon et al., 2018; Gray et al., 2019). Therefore, the clinical translation for stimuli-responsive methods still needs a lot of further investigations. Third, large-scale production of nanoparticles with consistent quality control is an underexplored issue; currently, most publications focus on the laboratory scale.

Moreover, nanotoxicity is a major issue in developing drug delivery systems and is highly related to nanomaterials. The success of many marked nanomedicines has eased the concerns. Liposomes and polymeric micelles have been demonstrated to be highly druggable and biocompatible. However, the non-biodegradable nanoparticles, such as inorganic nanoparticles, should be strictly restricted to therapeutic purposes due to their poorly documented safety.

Despite the difficulties, nanomedicine has achieved significant development, represented by dozens of products approved for market and hundreds in clinical trials, as well as thousands under preclinical research. A recent milestone success is Vyxeos (the liposome-encapsulated combination of daunorubicin and cytarabine) approved by FDA for acute myeloid leukemia (Alfayez et al., 2020). With the achievement of data-driven research methods including machine learning and artificial intelligence, nanoinformatics serves as a useful tool for nanomedicine design, which can manage and integrate various information including nanomaterials, manufacturing procedures, and nanotoxicity for computational prediction and design (Maojo et al., 2012; Sadan et al., 2018).

Last but not least, the availability and affordability of nanomedicines are important for society. In general, availability and affordability have been continuously improved. For example, the monthly cost of doxorubicin is $1,086 compared to $2,311 of its liposomal formulation (Bach and Elkin, 2020). Generic liposomes have further improved affordability.

It is expected that, with the development of nanoscience, nanotechnology-based HDACi will become a “magic bullet” against solid tumors.

## Author Contributions

BT wrote the draft. TL edited the manuscript. MZ and YH finalized the manuscript.

## Conflict of Interest

The authors declare that the research was conducted in the absence of any commercial or financial relationships that could be construed as a potential conflict of interest.
